# Relationship between triglyceride glucose index, retinopathy and nephropathy in Type 2 diabetes

**DOI:** 10.1002/edm2.151

**Published:** 2020-08-19

**Authors:** Sangeetha Srinivasan, Pallavi Singh, Vaitheeswaran Kulothungan, Tarun Sharma, Rajiv Raman

**Affiliations:** ^1^ Vision Research Foundation Chennai India; ^2^ Shri Bhagwan Mahavir Vitreoretinal Services Medical Research Foundation Chennai India

**Keywords:** diabetes, nephropathy, retinopathy, TyG index

## Abstract

**Aims:**

To explore the relationship between TyG index, diabetic retinopathy (DR) and nephropathy.

**Methods:**

This was a cross‐sectional observational study that examined 1413 subjects with type 2 diabetes (both known and newly diagnosed). Subjects underwent a detailed standard evaluation to detect diabetic retinopathy (fundus photography) and nephropathy (defined as urinary albumin excretion ≥ 30 mg/24 h). The TyG index was calculated as ln (fasting triglycerides (mg/dL) × fasting glucose (mg/dL)/2) and stratified into 4 quartiles (TyG‐Q). The baseline characteristics of the study population in the four TyG‐Q (Q1 (≤7.3) n = 349, Q2 (>7.3 to ≤ 7.5) n = 358, Q3 (>7.5 to ≤ 8.0) n = 354, and Q4 (>8.0) n = 352) were analysed. Variables associated with the presence of DR and nephropathy were assessed using a stepwise binary logistic regression analysis.

**Results:**

The presence of DR was associated with higher TyG index (OR = 1.453, *P* =.001) and longer duration of diabetes (OR = 1.085, *P* < .001). The presence of nephropathy was associated with a higher TyG index (OR = 1.703, *P* < .001), greater age (OR = 1.031, *P* < .001), use of insulin (OR = 1.842, *P* = .033), higher systolic BP (OR = 1.015, *P* < .001), and the presence of DR (OR = 3.052, *P* < .001). Higher TyG‐Q correlated with the severity of DR (*P* = .024), presence of nephropathy (*P* = .001), age (*P* < .001) and diastolic blood pressure (*P* = .006).

**Conclusions:**

A higher TyG index is associated with the presence of retinopathy and nephropathy in individuals with diabetes and could be used for monitoring metabolic status in clinical settings.

## Graphical abstract

Previous studies have demonstrated that the triglyceride glucose (TyG) index is a novel marker for metabolic disorders. We have shown that the TyG index is associated with diabetic retinopathy and nephropathy and may benefit from studying this as a potential marker in the incidence and progression of microvascular complications of diabetes.

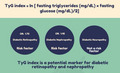

**What is already known?**

- The triglyceride glucose (TyG) index has been demonstrated as a novel marker for insulin resistance and cardiovascular disease. Both, glycaemic control and serum triglycerides are related to microangiopathy.
- The correlation with TyG index with diabetic retinopathy or nephropathy has not been explored.

**What this study has found?**

- We aimed to study the relationship between TyG index and diabetic retinopathy (DR), diabetic neuropathy and diabetic nephropathy.
- We observed that TyG index is associated with diabetic retinopathy as well as nephropathy.

**What are the clinical implications of the study?**

- TyG index could be used for monitoring metabolic status in clinical settings.

## 1 INTRODUCTION

Vascular complications are the leading cause of mortality and morbidity in type 2 diabetes, affecting smaller and larger blood vessels.^1^, ^2^ Microvascular complications may include but not limited to diabetic retinopathy (DR), diabetic neuropathy and diabetic nephropathy, while macrovascular complications include diseases of the coronary, peripheral and cerebral arteries. An optimal control of serum glucose has been the mainstay in the prevention of microvascular and macrovascular complications of diabetes. Nevertheless, abnormal plasma triglycerides have been associated with metabolic disorders and cardiovascular disease due to their interaction with raised glucose levels in fat, muscle and beta cells of the pancreas.^3^ We previously reported that the poor control of serum triglycerides is associated with progression to proliferative DR (PDR).^4^ Likewise, studies have shown that higher triglycerides predict future risk of albuminuria progression in patients with diabetes.^5^ Wiggin et al^6^ found that in patients with mild to moderate diabetic neuropathy, elevated triglyceride levels correlated with progressive myelinated fibre density loss which was independent of disease duration, age and glycaemic control. In effect, a product of fasting triglyceride and glucose (called TyG index) has been proposed and utilized to identify metabolically unhealthy individuals.^7^ The triglyceride glucose (TyG) index has been demonstrated as a novel marker for its association with insulin resistance and the risk of cardiovascular disease.^1^, ^8^, ^9^ However, there is no study demonstrating the relationship between TyG index and microangiopathy in diabetes. Therefore, we aimed to study the relationship between TyG index, diabetic retinopathy (DR), diabetic neuropathy and diabetic nephropathy.

## 2 METHODS

Study participants were recruited from the Sankara Nethralaya Diabetic Retinopathy Epidemiology and Molecular Genetic Study (SN‐DREAMS‐1). The study design and research methodology are described in detail elsewhere.^10^ In summary, the study area was the Chennai metropolis with a population of 4.3 million distributed in 155 divisions of 10 zones. As a sample, a total of 5999 subjects selected from the general population aged 40 years or above were enumerated; multistage stratified random sampling was performed on the basis of economic criteria. The data were compared between responders (1563 who visited the base hospital) and nonresponders (253 who did not visit the base hospital) with regard to mean age, gender, diabetes status and mean fasting blood sugar levels. No differences were observed. Of the 5999 subjects enumerated, 1413 persons identified with diabetes, as per World Health Organization criteria (both known and newly diagnosed), were examined for the study (96.20% response rate for first fasting blood sugar estimation, 85.60% response rate for base hospital examination, 8.7% turned out as nondiabetic after second blood sugar and 0.78% of retinal images were nongradable).

### 2.1 Definitions of biochemical variables

After 8 hours of overnight fasting, a blood sample was taken for estimating the plasma glucose and serum lipids. For those with provisional diabetes, confirmation of diabetes was done by re‐estimation of the fasting blood glucose by enzymatic assay; glucose was oxidized by glucose oxidase and produced gluconate and hydrogen peroxide, which was then analysed photometrically. Biochemical analysis was done on a Merck Microlab 120 semiautomated analyzer (Merck & Co., Inc, Whitehouse Station, NJ). The total serum cholesterol (cholesterol oxidase‐peroxidase [CHOD‐POD] method), HDL (after protein precipitation CHODPOD method) and serum triglycerides (CHOD‐POD) were estimated. The LDL cholesterol was calculated using the modified Friedewald formula for the Indian population.

The TyG index was calculated as ln[fasting triglycerides (mg/dL) × fasting glucose (mg/dL)/2].^7^

Patients were considered to have ‘newly diagnosed diabetes’ if the fasting blood glucose level was ≥ 110 mg/dL on two occasions.^11^ Patients were considered to have ‘known diabetes’ if they were using hypoglycaemic drugs, either oral or insulin, or both.

### 2.2 Diabetic retinopathy assessment

All patients had their retina photographed using the 45° four‐field stereoscopic digital photography (Carl Zeiss Fundus Camera, Visucamlite, and Jena, Germany) after pupillary dilatation. The presence and the severity of DR were noted based on the modified Klein classification (Modified Early Treatment Diabetic Retinopathy Study scales).^12^ This grading was done by two independent observers in a masked fashion; the grading agreement was high (*k* = 0.83).

### 2.3 Diabetic nephropathy assessment

Albuminuria estimation was done by a semi‐quantitative procedure (Bayer Clinitek 50 Urine Chemistry Analyzer) with the first morning urine sample. The patient was considered to have normoalbuminuria, if urinary albumin excretion (UAE) was < 30 mg/24 hour; microalbuminuria, if UAE was 30‐300 mg/24 hours; and macroalbuminuria, if UAE was > 300 mg/24 hours.^10^

### 2.4 Diabetic neuropathy assessment

Diabetic neuropathy assessment was done by measuring vibration perception threshold (VPT) using a sensitometer. The VPT was measured by a single observer by placing the biothesiometer probe perpendicular to the distal plantar surface of the great toe of both legs. The VPT was measured at a voltage level when patient felt the first vibration sensation. The mean VPT measure of three readings of both legs was considered for the analysis. Diabetic neuropathy was considered as present if the VPT value was ≥ 20 V.^10^

### 2.5 Statistical analysis

Continuous variables were assessed for normality of distribution. Inter‐group comparisons were assessed using ANOVA tests and were corrected for multiple testing using Tukey's method. Variables associated with the presence of DR and albuminuria were assessed using a stepwise binary logistic regression analysis. Statistical analysis was performed using the SPSS statistical package, version 25.0 (SPSS Inc, Chicago, IL, SPSS). *P* values <0.05 were considered statistically significant. Variables that were significant (*P* < .05) on univariate analysis were entered into the stepwise logistic regression.

## 3 RESULTS

The mean (SD) age of participants was 56.3 (10) years (range: 40‐85 years). The mean duration of diabetes was 5 (6) years (range: 0‐45 years). The difference in TyG between known (n = 1165) and newly detected (n = 248) diabetes was calculated. The mean (SD) was 7.4 (0.7) and 7.7 (0.6) for the known diabetes and newly detected diabetes groups, respectively (*P* < .001).

The TyG index was stratified into 4 TyG index quartiles (TyG index‐Q) namely ≤ 7.3, >7.3 and ≤ 7.5, >7.5 and ≤ 8.0, >8.0, based on data from current study participants. Table 1 shows baseline patient characteristics in the four TyGI‐Q. TyGI‐Q differed with age (*P* < .001), diastolic blood pressure (*P* = .006), fasting blood glucose (*P* < .001), serum total cholesterol (*P* < .001), high‐density lipoprotein (*P* < .001) and the use of alcohol (*P* = .003) and HbA_1c_ levels (*P* < .001). However, smoking (*P* = .111) and the use of insulin (*P* = .304) had no significant difference in the TyG index‐Q. The correlation between TyG and HbA_1c_ was analysed (*r* = 0.419, *P* < .001). In addition, HbA_1c_ and fasting blood sugar were correlated, *r* = 0.647, *P* < .001. Since HBA_1c_ was also correlated to fasting blood sugar, we did not include this in the statistical models.

**TABLE 1:** Baseline characteristics in the triglyceride index quartiles

Triglyceride index quartiles	A (≤7.3) n = 349		B (>7.3 to ≤ 7.5) n = 358		C (>7.5 to ≤ 8.0) n = 354		D (>8.0) n = 352		*P*
	Mean or n	SD/%	Mean or n	SD/%	Mean or n	SD/%	Mean or n	SD/%	
Age (y)	57.8	10.8	57.8	10.2	55.4	9.4	54.3	9.3	**<.001** **#**
Men	195		182		175		197		.181
Women	154		176		179		155		
BP systolic (mm Hg)	138	20	138	20	140	20	140	22	.69
BP diastolic (mm Hg)	81	12	81	11	83	12	83	11	.006*
Weight (Kg)	63.6	12.3	63.6	10.5	63.8	10.8	64.2	11.5	.898
Height (cm)	158.5	9.0	158.5	8.3	158.4	8.7	158.9	9.3	.859
BMI	18.6	11.8	20.7	10.8	20.6	33.6	20.7	11.6	.385
Waist circumference (cm)	90.8	10.5	91.3	9.7	91.4	9.7	91.8	9.4	.591
Hip circumference (cm)	101.0	12.3	100.7	10.9	101.2	9.3	100.3	9.6	.682
Waist/hip ratio	0.9	0.1	0.9	0.1	0.9	0.1	0.9	0.1	.14
FBS (mg%)	101.3	29.9	127.5	38.1	159.5	50.7	204.8	63.2	**<.001**
HbA1c (%)	7.1	1.8	7.6	1.9	8.5	2.1	9.5	2.1	**<.001**
Serum TC (mg%)	168.2	36.8	183.9	37.0	191.7	39.5	202.1	41.9	<.001**
Serum HDL (mg%)	41.4	10.8	39.9	9.9	38.6	9.7	37.0	9.9	**<.001##**
Ratio HDL/TC	0.2	0.1	0.2	0.1	0.2	0.0	0.2	0.1	**<.001**
Alcohol use									
Never	284	81.4%	296	82.7%	269	76.0%	254	72.2%	**.003**
Occasional	48	13.8%	40	11.2%	63	17.8%	57	16.2%	
Regular	16	4.6%	20	5.6%	18	5.1%	38	10.8%	
Heavy drinker	1	0.3%	2	0.6%	4	1.1%	3	0.9%	
Nonsmoker	283	81.1%	292	81.6%	285	80.5%	276	78.4%	.111
Current smoker	27	7.7%	34	9.5%	46	13.0%	42	11.9%	
Ex‐smoker	39	11.2%	32	8.9%	23	6.5%	34	9.7%	
Insulin use (yes)	17	7.7%	21	8.70%	14	6.40%	25	11.30%	.304
Insulin use (no)	205	92.3%	221	91.30%	205	93.60%	197	88.70%	

Significant *P* values are indicated in bold.

Abbreviations: BMI, body mass index; BP, blood pressure; FBS, fasting blood sugar; HDL, high‐density lipoprotein; TC, total cholesterol.

Post hoc comparisons: #A Vs C & D, *A Vs D, **all comparison significant, ##A Vs C & D.

The mean (SD) for nephropathy present was 7.6 (0.6) and 7.4 (0.7) for nephropathy absent, *P* < .001. The mean (SD) for DR present was 7.6 (0.7) and 7.4 (0.7) for DR absent, *P* = .005. Furthermore, Table 2 shows diabetic microangiopathy distribution in the four TyGI‐Q. The TyGI‐Q were significantly different with the severity of DR (*P* = .024), albuminuria (*P* = .001), but not in the presence of diabetic neuropathy (*P* = .35). Among those with DR (n = 255), 81 (31.7%) had diabetic macular oedema (DME). The mean (SD) of TyG index was 7.7 (0.6) in those with DME and 7.5 (0.7) in those with no DME, *P* = .196. Proportion of subjects with DME in the four quartiles is shown in Table 2.

**TABLE 2:** Diabetic microangiopathy characteristics in the triglyceride index quartiles

Triglyceride index quartiles	A (<= 7.3) n = 349		B (>7.3 to <= 7.5) n = 358		C (>7.5 to <= 8.0) n = 354		D (>8.0) n = 352		*P*
	n	%	n	%	n	%	n	%	
Diabetic Retinopathy (DR)									
No DR	299	85.7%	297	83.0%	291	82.2%	271	77.0%	**.024**
Mild NPDR	26	7.4%	36	10.1%	29	8.2%	36	10.2%	
Moderate NPDR	15	4.3%	18	5.0%	21	5.9%	34	9.7%	
Severe NPDR	2	0.6%	1	0.3%	7	2.0%	8	2.3%	
PDR	7	2.0%	6	1.7%	6	1.7%	3	0.9%	
Any DR	50		61		63		81		
DME present	13		16		26		26		.235
Albuminuria									
<30 mg/24 h	306	87.7%	295	82.40%	286	80.80%	262	74.40%	**.001**
30‐300 mg/24 h	34	9.7%	56	15.60%	57	16.10%	79	22.40%	
>300 mg/24 h	9	2.6%	7	2.00%	11	3.10%	11	3.10%	
Overall albuminuria	43		63		68			90	
Diabetic Neuropathy									
No	273	78.7%	298	83.90%	282	80.60%	283	81.30%	.35
Yes	74	21.3%	57	16.10%	68	19.40%	65	18.70%	

Significant *P* values are indicated in bold.

Abbreviations: NPDR, nonproliferative DR; PDR, proliferative DR.

Table 3 shows the variables associated with the presence of DR, and with the presence of abnormal albuminuria. The presence of DR was associated with higher TyG index (OR = 1.453, *P* = .001) and longer duration of diabetes (OR = 1.085, *P* < .001), use of insulin (OR = 3.459, *P* < .001), and men (OR = 1.459, *P* = .018). The presence of abnormal albuminuria (defined as urinary albumin excretion ≥ 30 mg/24 hours) was associated with a higher TyG index (OR = 1.703, *P* < .001), greater age (OR = 1.031, *P* < .001), use of insulin (OR = 1.842, *P* = .033), higher systolic BP (OR = 1.015, *P* < .001) and the presence of DR (OR = 3.052, *P* < .001).

**TABLE 3:** Stepwise binary logistic regression for presence of diabetic retinopathy and nephropathy

	OR	95% CI		*P*
Presence of Diabetic Retinopathy^a^				
Triglyceride Index	1.453	1.171	1.803	.001
Men	1.459	1.111	2.000	.018
Duration of diabetes	1.085	1.061	1.110	<.001
Insulin treatment	3.459	1.991	6.009	<.001
Microalbuminuria 30‐300	2.515	1.746	3.623	<.001
Macroalbuminuria > 300	6.873	3.304	14.297	<.001
Presence of Diabetic Nephropathy^b^				
Triglyceride Index	1.703	1.369	2.117	<.001
Age	1.031	1.015	1.048	<.001
Insulin treatment	1.842	1.050	3.232	.033
Systolic BP	1.015	1.008	1.022	<.001
Presence of DR	3.052	2.191	4.251	<.001

Abbreviation: DR, diabetic retinopathy

^a^ Adjusted for age, smoking, blood pressure.

^b^ Adjusted for gender, smoking, duration of diabetes.

Table 4 shows the variables associated with the presence of microalbuminuria and macroalbuminuria. The presence of microalbuminuria (OR = 2.515, *P* < .001) and macroalbuminuria (OR = 6.873, *P* < .001) were associated with greater odds of having DR.

**TABLE 4:** Stepwise binary logistic regression for presence of microalbuminuria and macroalbuminuria

	OR	95% CI		*P*
Presence of microalbuminuria^a^				
Triglyceride Index	1.748	1.390	2.199	<.001
Age	1.029	1.012	1.047	<.001
Diastolic BP	1.014	1.006	1.021	<.001
Presence of DR	2.721	1.919	3.860	<.001
Presence of macroalbuminuria^a^				
Diastolic BP	1.02	1.01	1.04	<.001
Presence of DR	8.29	4.12	16.68	<.001

Abbreviations: BP, blood pressure; DR, diabetic retinopathy.

^a^ Adjusted for triglyceride index, gender, smoking, duration of diabetes, insulin treatment, diastolic BP.

## 4 DISCUSSION

The TyG index has been examined in relation to metabolic disorders including diabetes and cardiovascular diseases in previous studies.^13^, ^14^, ^15^, ^16^, ^17^, ^18^, ^19^ The current study examined the TyG index in relation to diabetic retinopathy, neuropathy and nephropathy, an association which has not been reported in literature.

It was observed that TyG index is independently associated with the presence of DR when adjusted for age, smoking and blood pressure. Similarly, a higher TyG index is associated with about twice the odds of having micro‐ and macroalbuminuria. However, TyG index is not related to diabetic neuropathy in our study.

We found that the proportion of those with and without DR differed significantly among the four quartiles. The formula for assessing TyG index incorporates both fasting glucose and triglyceride levels into consideration. We observed that the fasting blood glucose is higher with higher quartiles of TyG indices. The association between fasting blood glucose and DR is well established. Previous study reported increased prevalence of DR with fasting blood glucose greater than 7.03 mmol/L and for a HbA_1c_ more than 6.4% (46 mmol/mol).^20^ This further confirms the association between higher fasting blood glucose, triglyceride and DR. It has been proposed that the triglycerides and triglyceride‐rich lipoproteins are causal factors related to cardiovascular diseases^21^ and microvascular complications of diabetes.^22^ Although the exact mechanism is not known, triglyceride levels are proposed to be linked to microvascular complications by means of lipid peroxidation^23^ and endothelial dysfunction, associated with retinal and renal complications in diabetes.^24^ In addition, inflammatory markers including tumour necrosis factor‐α, interleukins, leucocytes and fibrinogen may cause atherosclerotic plaque been proposed to play a crucial role in metabolic syndrome and related disorders,^25^, ^26^ and may play a similar role in the retina.

The current study has shown that fasting glucose and triglycerides are independently associated with diabetic nephropathy. In addition, the presence of abnormal albuminuria (defined as urinary albumin excretion ≥ 30 mg/24 hours) was associated with a higher TyG Index. It is well known that DR and albuminuria are associated with each other. We have previously reported in the same cohort that^27^ every 6th individual with type 2 diabetes has albuminuria and that they are twice as likely to have DR in the presence of microalbuminuria and six times as likely to have DR in the presence of macroalbuminuria. In the current study, when adjusted for TyG index, we observed that those with DR are three times likely to have albuminuria than those with no DR. Microalbuminuria and degree of retinopathy are correlated, and this correlation can be explained by the common mechanism involved in tissue damage. In addition to blood glucose level and blood pressure, it has been speculated that the use of insulin can have a role in nephropathy^28^ which was also similar to that observed in the current study. Deckert et al^29^ proposed that in microalbuminuria, endothelial cell damage causes a lowering of lipoprotein lipase levels at the endothelium, leading to widespread vascular damage, causing an increase in the concentration of plasma triglycerides leading to hypertriglyceridaemia.

Likewise, Kim et al^30^ reported that fasting plasma level of insulin and systolic blood pressure have independent correlation with microalbuminuria. An elevated plasma glucose levels, excess abdominal obesity, a higher blood pressure and abnormal lipid levels may co‐exist, a collective term called metabolic syndrome (MS).^31^, ^32^ Our previous report from the same population reported a prevalence of metabolic syndrome to be 73.3%.^33^ The prevalence of DR in people without and with MS (21.3% and 16.9%, *P* =.057), and the prevalence of nephropathy (20.5% and 18.0%, respectively) (*P* =.296) did not differ. However, in the current study, we found a difference in DR and in diabetic nephropathy with respect to TyG index. In the current population, TyG index appears to be a better predictor for diabetic complications comparing to the aforementioned factors that also play a role in MS.

We found no correlation between TyG index and diabetic neuropathy. Kwai et al^34^ also found that there was no association between changes in axonal function and triglyceride levels in a cohort of type 2 diabetic patients. It is likely that other mediating factors apart from hypertriglyceridaemia and hyperglycaemia may be involved in mediating diabetic neuropathy. Potential candidates may include increased circulating inflammatory markers including NF‐κB, which is involved in altered thermonociception.

Our study was conducted in Indian population in Tamil Nadu, India. We believe that our study results may not be applicable to the other ethnic groups because Indians (Asian Indians) possess distinctive characteristics of obesity, a higher abdominal fat, and fat deposition in abdomen, liver and muscle. Obesity which indicates ‘generalized obesity’ or excess body fat can be assessed by BMI. Abdominal adiposity could be assessed by waist circumference or waist‐to‐hip ratio.^35^ South Asians have greater predisposition to abdominal obesity and visceral fat, which is attributed to the so‐called ‘Asian Indian phenotype’ characterized by increased waist circumference despite lower BMI.^36^ Based on percentage body fat and morbidity data, normal BMI is narrower and lower in Asian Indians than in white Caucasians.^37^ Therefore, these results may not be applicable to other ethnic groups.

Insulin resistance (IR) is the decreased sensitivity of tissues to insulin and is a predisposing factor for hyperglycaemia, higher blood pressure and dyslipidaemia. The clamp method which is the accepted gold standard for direct measurement of IR is impractical in clinical settings as it requires sophisticated equipment.^38^ Therefore, many other surrogate methods have been explored to indirectly measure IR. One of the most widely used techniques is the homeostatic model assessment of IR (HOMA‐IR). HOMA‐IR is calculated based on the measurement of fasting glucose and insulin levels. However, insulin levels show a wide range of intra‐ and inter‐subject variability, and also, the measurement of insulin levels is not standardized. Therefore, attempts have been made to identify several other parameters as a measure of IR. Lipids have been explored as a possible index to determine insulin action to assess IR. Higher triglyceride levels are related to poor glucose metabolism in muscles aligning with the proposition that triglyceride elevation in serum and tissue is related to decreased insulin sensitivity, even though the exact mechanism is unclear. In 2010, Guerrero et al^7^ showed that the product of TG and glucose in plasma, the so‐called triglycerides and glucose index (TyG), could be a useful estimate of IR. They assessed individual indices such as BMI, waist circumference, FBS, triglycerides, insulin, Homa‐IR index, total glucose metabolism and TyG‐index to recognize IR in subjects who were healthy, obese, had prediabetes and diabetes. The authors observed that the TyG index closely mirrored the glucose clamp technique in the assessment of insulin with a high sensitivity (96.5%) and specificity (85.0%) in comparison to the hyperinsulinaemic‐euglycaemic clamp technique. The TyG index therefore has an advantage over other indices to assess insulin resistance.^39^

The mean (SD) of TyG index was 7.4 (0.7) and 7.7 (0.6) for the known diabetes and newly detected diabetes groups, respectively (*P* < .001). This only means that those with known diabetes must be on medication or some form of control and, therefore, may reflect a slightly lower TyG index values than the newly detected diabetes patients.

There were no significant differences in TyG index between those with and without diabetic macular oedema. One explanation could be due to small number of participants with diabetic macular oedema. Another likely explanation could be inconsistent association or differential association between the type of serum lipids and diabetic macular oedema. We previously reported that high serum low‐density lipoprotein, non‐high‐density lipoprotein, and high cholesterol ratio were related to non‐clinically significant macular oedema, and while high serum total cholesterol was related to clinically significant macular oedema.^40^ In addition, we also reported that total cholesterol was associated with the incidence and all lipid types were associated with progression to sight‐threatening retinopathy.^4^

The strength of our study is that it is a large population‐based study. The photographic standard way of grading diabetic retinopathy is another strength of this study. The number of patients with DR was not high. Nevertheless, we observed a significant association between the presence of DR and TyG index. There were several limitations in the present study. Firstly, the measurements of TG and fasting glucose had unavoidable intra‐individual biological variation. We did not study insulin resistance. Moreover, other confounding factors such as exercise habit, nutritional status and cardiorespiratory status were not included in the model. Although an association was observed between TyG‐Q and the severity of DR (Table 2), there were a small number of participants with proliferative DR in all four quartiles. Therefore, the results must be interpreted with this observation in mind.

In conclusion, we found that a higher triglyceride glucose index is independently associated with retinopathy and nephropathy in individuals with diabetes. TyG may offer some clue about the presence and severity of DR, but whether it can be used as replacement to the existing risk factors is still questionable. But in the Indian population, where anaemia is a problem, anaemia can alter HbA_1c_ levels.^41^ In this instance, TyG may be helpful in the assessment of DR. Further studies may be required to explore whether TyG index has a role in the incidence and progression of microvascular complications of diabetes.

## CONFLICT OF INTEREST

Nothing to declare.

## AUTHORS' CONTRIBUTIONS

RRN and TS conceptualized the study; SS involved in data curation and analysis; RRN, TS and VK investigated the study and designed the methodology; SS, RRN and TS provided the resources and software; RRN validated the study; SS, PAL and RRN wrote the original draft of the manuscript; and SS and RRN wrote, reviewed and edited the manuscript.

## ETHICAL APPROVAL

The study was approved by the Institutional Review Board, Vision Research Foundation, Chennai, India. (Grant number 10‐2003‐P) Subjects provided written informed consent, and the study was conducted according to the tenets of the Declaration of Helsinki.

## Data Availability

The data that support the findings of this study are available from the corresponding author upon reasonable request.
